# Identification of *Diatraea* spp. (Lepidoptera: Crambidae) based on cytochrome oxidase II

**DOI:** 10.1371/journal.pone.0184053

**Published:** 2017-09-05

**Authors:** Gloria Patricia Barrera, Laura Fernanda Villamizar, Carlos Espinel, Edgar Mauricio Quintero, Mariano Nicolás Belaich, Deisy Liseth Toloza, Pablo Daniel Ghiringhelli, Germán Vargas

**Affiliations:** 1 Centro de investigación Tibaitatá, Corporación Colombiana de Investigación Agropecuaria CORPOICA, Mosquera, Cundinamarca, Colombia; 2 AgResearch Ltd. Lincoln Research Centre, Christchurch, New Zealand; 3 Centro de investigación de la caña de azúcar de Colombia (CENICAÑA), Calle 58 norte No. 3BN-110. Cali, Colombia; 4 Laboratorio de Ingeniería Genética y Biología Celular y Molecular—Área Virosis de Insectos (LIGBCM—AVI), Dto. de Ciencia y Tecnología, Universidad Nacional de Quilmes, Roque Saenz Peña 352, Bernal, Provincia de Buenos Aires, Argentina; Chang Gung University, TAIWAN

## Abstract

*Diatraea* spp. (Lepidoptera: Crambidae) are a group of insects that are agriculture pests in many economically relevant crops such as sugarcane, sorghum, corn and rice. Recognized species for this genus respond differentially to natural enemies used in their biological control, emphasizing the importance of species in a regional approach. Currently, identification is based on the male genitalia. However, the availability of specimens collected from field and subjectivity based on the character recognition can seriously hamper species identification, and therefore result in inadequate pest management. To overcome this, individuals of *Diatraea* spp. preliminarily classified male genitalia and obtained from reared conditions and the field (both derived from natural populations occurring in Colombia) were analyzed using genitalic morphometry and molecular biology specifically using a fragment of the *cytochrome oxidase subunit* II (CO II) mitochondrial gene. Although morphometric analysis did not show any overriding results regarding genitalia morphology, the bioinformatics analyses of CO II sequences resulted in an adequate classification of the individuals within the recognized species. It also, revealed that the occurrence of clades associated with geographical distribution may be associated with cryptic species. The latter was also confirmed by a Single-Strand Conformation Polymorphism (SSCP) methodology evaluating the same fragment of CO II. This experimental approach allows properly recognizing each species and in consequence is proposed as an effective tool in *Diatraea* species identification.

## Introduction

Stem borers of the genus *Diatraea* (Lepidoptera: Crambidae) are major pests in the Americas because larvae cause serious damages in important crops including sugarcane, sorghum, corn and rice [1–4]. In young plants of sugarcane, the attack may compromise the meristematic tissue producing the symptom known as "dead heart" and the death of the inner whorls of leaves. Later in the development of the crop, the direct damage in the stalks by tunneling and breaking thorough the tissues interferes with the movement of nutrients, the distribution of photosynthates and tends to increase the level of fiber in the affected stalk decreasing its weight and value [3, 5]. *Diatraea* has at least 41 recognized species in the Western Hemisphere [in Colombia *D*. *saccharalis* (Fabricius), *D*. *indigenella* Dyar & Heinrich, *D*. *lineolata* (Walker), *D*. *tabernella* Dyar and *D*. *busckella* Dyar & Heinrich] *D*. *saccharalis* being the species with the widest distribution [6]. In fact, this insect can be found in sugarcane crops from USA to Argentina [7]. The high diversity of species suggests that the environment and host plants could induce the occurrence of cryptic species as reported in several geographical locations of America [8–11].

Historically, the identification of *Diatraea* species relies only on the dissection of adult male genitalia [6, 12–13]. However, different species are not distinguishable from each other based on external characters of either the adult or the larval stage [14], and recovering males for conclusive examination could be challenged by larval parasitism in field by either tachinid flies or *Cotesia flavipes* Cameron (Hymenoptera: Braconidae). In view of that, new approaches based on DNA techniques may be useful to recognize those species with little morphological divergence. Particularly, the use of the *cytochrome oxidase subunit* I and II (COI and II) mitochondrial genes have been of great help for genetic differentiation across several animal taxa [15–20]. Different efforts have been made on *Diatraea* spp. based on these sequences [9, 21–25] proposing an opportunity to have a more precise species identification.

Species identification in *Diatraea* plays an important role in the development of effective pest control strategies, due to the differential response to natural enemies. For example, the tachinid fly *Lydella minense* (Townsend) and *Trichogramma exiguum* Pinto and Platner (Hymenoptera: Trichogrammatidae) are used in biological control of the pest in Colombia, but the continuous release of *L*. *minense* in Colombian northern Cauca River Valley did not prevent a *D*. *tabernella* outbreak in the region as the tachinid shows a preference to parasitize larvae of *D*. *saccharalis* over those of *D*. *tabernella* [26].

Accurate pest species identification and their association with the main natural enemies exerting regulation on their populations is a requirement for sustaining biological control programs. Considering the above, the aim of this work was to evaluate different methods to precisely identify *Diatraea* species based on morphometric tools associated with genitalic morphometric analysis, complemented with an analysis of single-strand conformation polymorphism (SSCP) associated to *in vitro* amplified fragments of the mitochondrial CO II. In addition, the sequences corresponding to this gene were studied to infer phylogenetic relationships among *Diatraea* species and to discuss the occurrence of cryptic species.

## Materials and methods

### Insect source and rearing

Male adult insects of *Diatraea saccharalis* (n = 38), *D*. *indigenella* (n = 25), *D*. *tabernella* (n = 26) and *D*. *busckella* (n = 34) were obtained from insect colonies established at the Colombian Sugarcane Research Center (CENICAÑA) which were maintained in controlled conditions (24°C ± 2 and 77% ± 7 relative humidity). These colonies were periodically refreshed at least every two months with field individuals from different sugarcane plots in the Cauca River Valley (Colombia) and identified by male genitalia [6]. On the other hand, adult males of *D*. *centrella* (Möschler) (n *=* 15), *D*. *albicrinella* (Box) (n = 1*)* and individuals with undescribed characteristics, here labelled as DiatraeaSolisVargas05 (n = 5), were also recognized by genitalia, but collected in sugarcane crops from Caquetá (Colombia), while additional individuals of *D*. *saccharalis* (n = 4) were obtained from Cundinamarca (Colombia). Reared insects (*D*. *saccharalis*, *D*. *indigenella*, *D*. *busckella* and *D*. *tabernella*) were used to morphometric analysis of genitalia. Abdomens from field and reared insects were dissected and maintained in ethanol at 70% v/v in sterile distilled water to DNA extractions and then used for molecular analysis.

### Genitalia

Genitalia were obtained from male adults following reported methodology [27, 28]. Then, lateral tegumen lobes from at least 10 individuals from each reared species were registered by light micrographs (stereomicroscope Carl Zeiss Stemi™ DV4 adapted with a Zeiss Axio Cam™ ERc5s). Lobe dimensions were determined by using ZEN 2 imaging software© (Carl Zeiss Microscopy GmbH, 2011) and data were analyzed using one-way ANOVA and means separation using Least Significative Difference, LSD test (α = 0.05).

### DNA extraction and amplification

DNA of male insects were obtained of complete abdomen by using commercial kit (DNeasy Blood & Tissue Kit, QIAGEN) and quantified by spectrophotometry (Nanodrop 1000, Thermo-Fisher). Then, *in vitro* amplifications of CO II fragments were carried out by the Polymerase Chain Reaction method (PCR) in standard conditions (final volume 25 μL) using Taq polymerase (PROMEGA), 50 ng of template and the primers previously described and used in *Diatraea*: A-298 (5’-ATTGGACATCAATGATATTGA-3’) and B-tLYS (5’ GTTTAAGAGACCAGTACTTG-3’) [22, 23, 29]. For Sanger sequencing the primers were re-designed including universal sequences: M13 forward/A-298 (5'-GGTTTTCCCAGTCACGACATTGGACATCAATGATATTGA-3') and M13 reverse/BTLYS (5’-GGCAGGAAACAGCTATGACGTTTAAGAGACCAGTACTTG-3’). Thermal cycling was performed using the following conditions: 1 cycle at 95°C for 3 min; 34 cycles at 95°C for 10 s; 53°C for 45 s, and 72°C for 30 s; and one cycle at 72°C for 5 min. Aliquots of amplification products were resolved in 1% w/v agarose gel electrophoresis and later stained with SYBR-Safe (Invitrogen). The PCR products generated with M13 forward/A-298 and M13 reverse/BTLYS primers were sequenced using M13 universal oligonucleotides (Macrogen, Korea) and the other amplicons were used for SSCP methodology.

### Single-Strand Conformation Polymorphism (SSCP) methodology

The amplicons were prepared for SSCPs. To this, 1 μL of PCR products amplified using A-298/B-tLYS were mixed with 9 μL of sample buffer (95% v/v Formamide, 20mM EDTA, 0.05% w/v bromophenol blue and 0.05% w/v Xylene-cyanol, in distilled water), denatured at 95°C for 5 min and immediately stored in ice bath. Then, 1.5 μL of all samples were loaded in 6% non-denaturing polyacrylamide gel (49 acrylamide: 1 bis-acrylamide), and separated by electrophoresis for 4 hours at 600V (BioRad Sequi-Gen GT Nucleic Acid Electrophoresis Cell, 38 x 50 cm). Later, PAGEs were stained using and adapted silver nitrate method. Briefly, gels were fixed for 3 min (10% v/v ethanol, 1% v/v acetic acid in distilled water), oxidized for 3 min (1.5% v/v nitric acid in distilled water), stained for 20 min (0.1% w/v silver nitrate, 0.045% v/v formaldehyde in distilled water), revealed for approximately 5 min (3% w/v NaCO_3_, 0.02% v/v formaldehyde in distilled water), and the reactions were stopped with an acetic acid solution (5% v/v in distilled water). Results were documented by digital photography.

### Bioinformatics analyses

The sequences of partial CO II mitochondrial gene were manually inspected and used to infer phylogeny and evolutionary distances by Neighbor-Joining method using MEGA 6 [30] with the following set of parameters: Bootstrap with 1000 replicates; Model = Kimura 2-parameters; Substitutions to include = D: Transitions + Transversions; Rates among sites = uniform; Patterns among sites = Same (Homogeneous); and Gaps/Missing data = Pairwise deletion. GenBank accessions numbers included in the bioinformatic analyses were: MF379467 to MF379500 (*D*. *busckella*), MF379501 to MF379525 (*D*. *indigenella*), MF379526 to MF379563 (*D*. *saccharalis* 1), MF379564 to MF379567 (*D*. *saccharalis* 2), MF379568 to MF379593 (*D*. *tabernella*), MF379594 to MF379608 (*D*. *centrella*) and MF379609 to MF379614 (DiatraeaSolisVargas05). Additionally, the relative abundances of trinucleotides present in the amplicons were calculated to produce gene signatures for each *Diatraea* taxa. Then, the averaged values were compared among them to estimate distances [31]. Both distances calculated by Kimura 2-parameters and the relative abundance of trinucleotide values were diagrammed using Sigma Plot software v9.

The single-stranded DNA secondary structure predictions corresponding to DNA sequence consensus for each *Diatraea* species were done using the Mfold programme [32] with the following conditions: DNA free energy parameters with 1.0 M Na+ concentration and 0.0 M Mg2+, at 25°C [33].

## Results and discussion

### Morphometric analyses

**Fig 1:**
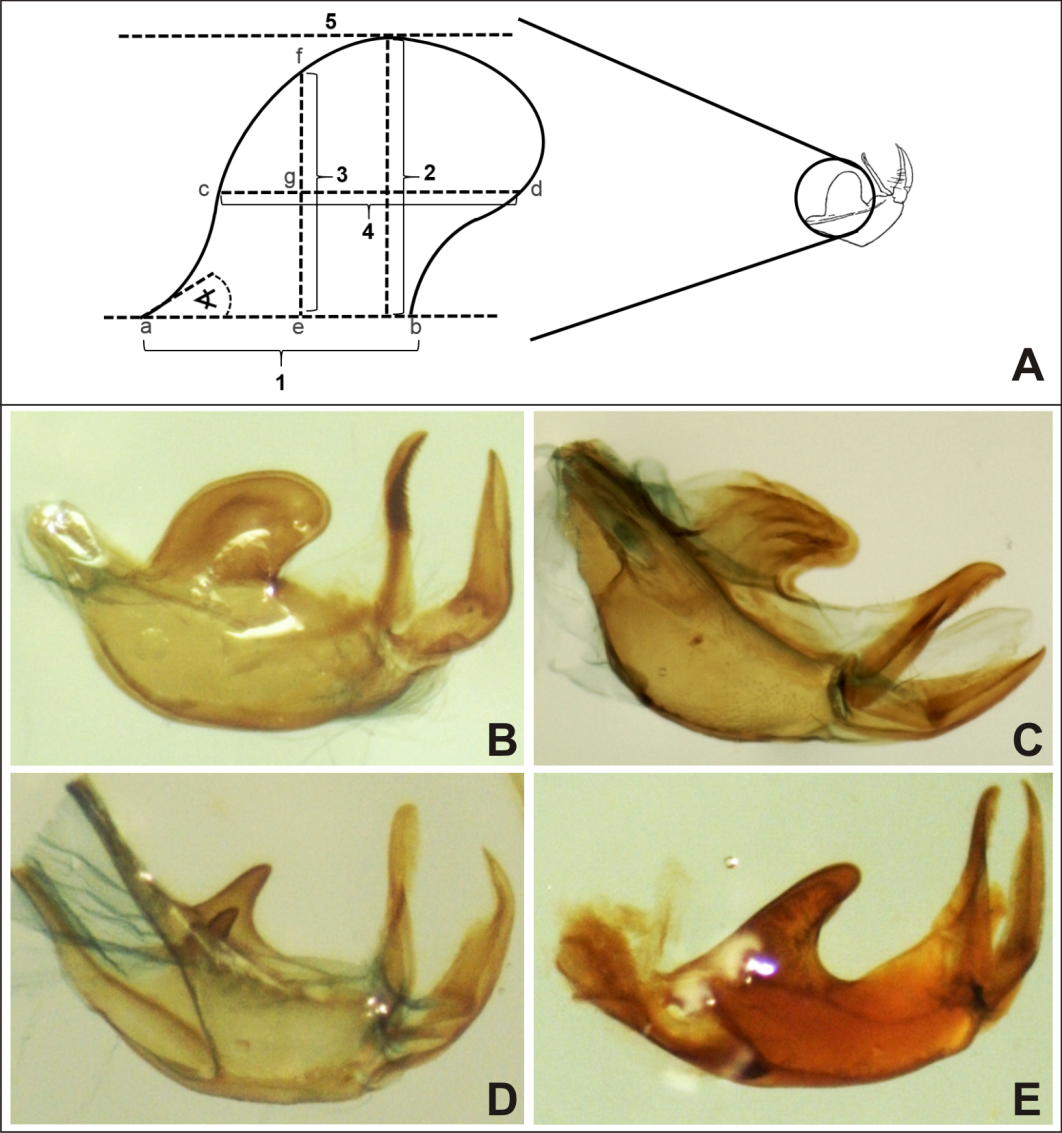
Morphometric analyses of the lateral lobe on the tegumen on *Diatraea* male genitalia. **A.** Representation of the measures estimated of the lateral lobes of the tegumen. 1. (a—b) Lobe base; 2. Maximum height of the lobe, from base (1) to the highest point, estimated by the tangent formed by tracing the parallel to the base (5); 3. (e—f) Midpoint lobe height, measured from the midpoint of the lobe base (e); 4. (c—d) Lobe width measured from midpoint of 3; Angle: formed by the tangent line at the point “a” to the lobe base (1). Figure of the tegumen (right), taken from Bleszynski [7]. **B.** Lateral lobes of the tegumen of male genitalia from *Diatraea saccharalis*. **C.** Lateral lobes of the tegumen of male genitalia from *Diatraea busckella*. **D.** Lateral lobes of the tegumen of male genitalia from *Diatraea indigenella*. **E.** Lateral lobes of the tegumen of male genitalia from *Diatraea tabernella*.

Currently the identification method for *Diatraea* species is based on morphological characteristics of adult male genitalia, and usually based on the lateral lobes of the tegumen [6]. However, this qualitative evaluation can be influenced by the experience of the observer and the possibility that an individual specimen may lack this character. To improve the genitalia observation methodology, a morphometric study on the lateral lobes of the tegumen of the male genitalia was performed in individuals of 4 species from laboratory colonies (Fig 1).

The values of each measure were averaged and compared among them to add quantitative data to the observations (**Table 1**).

**Table 1:** Average results of the different estimated dimensions of the lateral lobes of the tegumen of specimens from 4 species of *Diatraea*. Statistical comparisons were carried out independently for each dimension. Treatments with the different letters are significantly different according to LSD (95%). SD (Standard deviation); CV (coefficient of variation).

Dimension	Species	Mean value (μm)	SD	CV (%)
Base of the lobe	*D*. *busckella*	2217.69 ab	391.43	17.65
	*D*. *indigenella*	1624.10 c	360.33	22.19
	*D*. *tabernella*	2366.74 a	281.29	11.89
	*D*. *saccharalis*	2016.04 b	307.96	15.28
Maximum lobe height	*D*. *busckella*	1744.80 b	233.21	13.37
	*D*. *indigenella*	1087.63 c	283.66	26.08
	*D*. *tabernella*	1963.74 ab	204.06	10.39
	*D*. *saccharalis*	2143.46 a	323.63	15.10
Midpoint lobe height	*D*. *busckella*	1438.09 b	185.56	12.90
	*D*. *indigenella*	859.51 c	213.33	24.82
	*D*. *tabernella*	1401.05 b	175.01	12.49
	*D*. *saccharalis*	1662.67 a	320.38	19.27
Lobe width	*D*. *busckella*	1724.04 ab	243.99	14.15
	*D*. *indigenella*	634.00 c	136.87	21.59
	*D*. *tabernella*	1643.11 b	162.33	9.88
	*D*. *saccharalis*	1911.99 a	454.50	23.77
Angle	*D*. *busckella*	58.90 a	6.56	11.13
	*D*. *indigenella*	38.80 c	9.52	24.53
	*D*. *tabernella*	52.20 b	3.58	6.87
	*D*. *saccharalis*	61.80 a	4.87	7.88
Lobe area	*D*. *busckella*	2655.79 b	542.90	20.44
	*D*. *indigenella*	610.07 c	150.49	24.67
	*D*. *tabernella*	2878.34 b	532.51	18.50
	*D*. *saccharalis*	3764.42 a	725.19	19.26

*D*. *tabernella* presented the largest base of the lobe which was not significantly different from *D*. *busckella*, but was significantly larger (F = 9.07; df = 3; P = 0.0001) than that of *D*. *saccharalis* and *D*. *indigenella*. *D*. *saccharalis* and *D*. *tabernella* did not show differences in the maximum lobe height, but this dimension for *D*. *saccharalis* was significantly larger (F = 30.0; df = 3; P < 0.0001) than that of *D*. *busckella* and *D*. *indigenella*. When midpoint lobe height was measured, *D*. *saccharalis* presented the maximum value, which was statistically different from all other species (F = 21.8; df = 3; P < 0.0001), while *D*. *busckella* and *D*. *tabernella* were not different between them and same trend was determined for the lobe area (F = 101; df = 3; P < 0.0001).

*Diatraea saccharalis* and *D*. *busckella* did not show differences for lobe width and angle. *D*. *tabernella* presented a lobe width similar to *D*. *busckella* but significantly smaller than the obtained with *D*. *saccharalis* and significantly larger than *D*. *indigenella* (F = 42.3; df = 3; P < 0.0001). The lobe angle of *D*. *tabernella* was more acute than the angle for *D*. *saccharalis* and *D*. *busckella*, but wider than that of *D*. *indigenella* (F = 42.3; df = 3; P < 0.0001).

With the exception of *D*. *indigenella*, the lateral lobe of the tegumen of the species studied here presented similar dimensions, being evident that the main difference is not the size but the shape of this genital structure, which could be related to the lock-and-key hypothesis [34]. The Darwinian lock-and-key hypothesis explains genital divergence as a form of character displacement. However, there is little empirical or experimental evidence to support this assumption, and current understanding is that genitalic evolution is driven mostly by sexual selection [35–37].

For all dimensions of the lateral lobe of the tegumen, *D*. *indigenella* presented lower values than those obtained with the other species. In this sense, *D*. *indigenella* is then easily differentiated from the others, presenting a significantly smaller lobe showing a particular acute triangular shape (Fig 1D). It is important to mention that the high standard deviation calculated among individuals from the same species reveals high intraspecific variation and therefore, these analyses would not allow a proper species identification. However, it provides a quantitative approach that complements morphological observations of genitalia.

### Phylogenetic analyses

**Fig 2:**
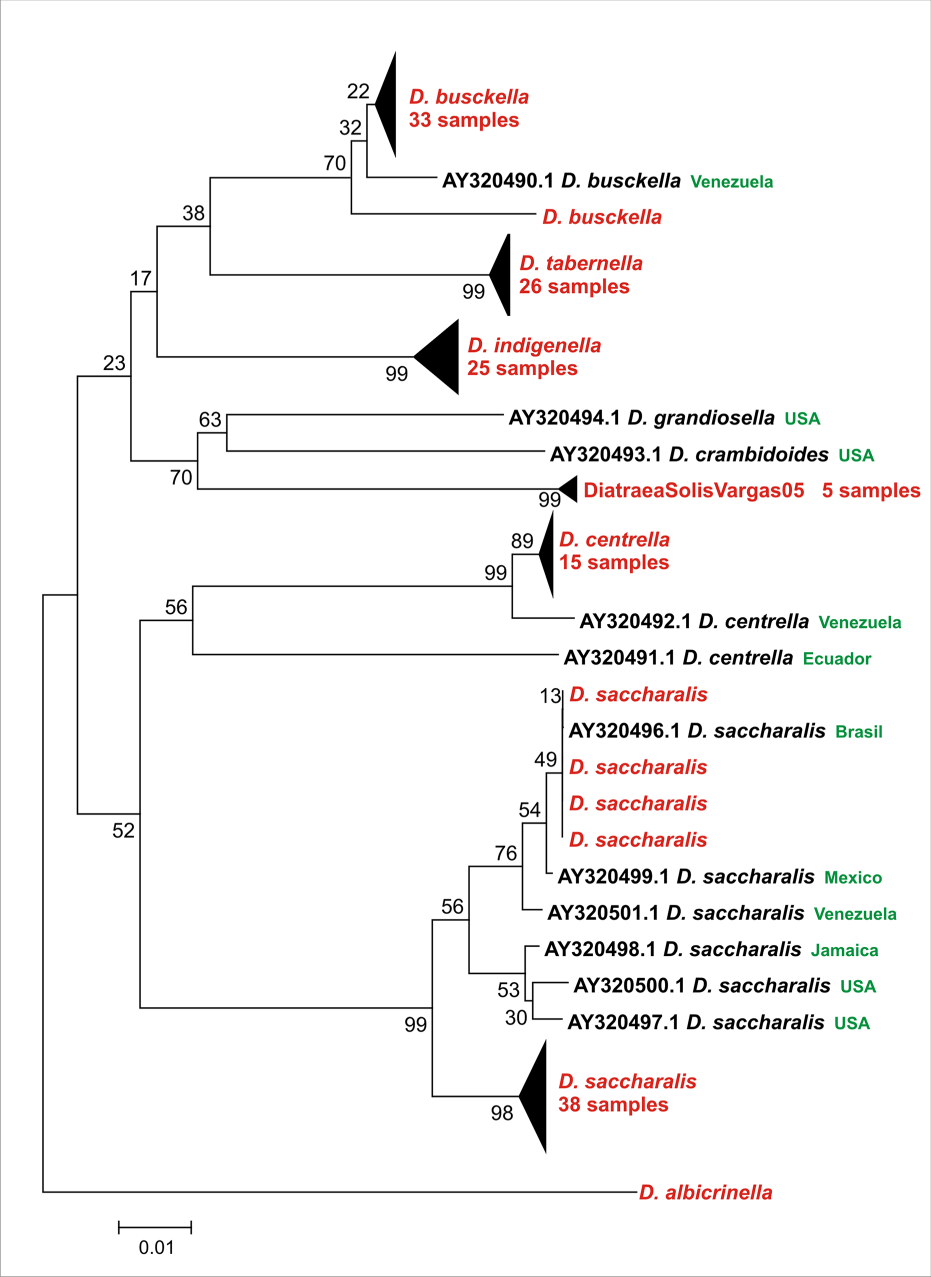
Neighbor-Joining phylogenetic tree. Cladogram generated using Kimura 2-parameter method. Numbers on nodes indicate their consistency expressed as percentages. GenBank sequences used [22] are referred with its accession numbers and are indicated in black letters. Besides, their geographical origins are indicated in green letter. Sequences obtained by this work are indicated in red. Some clades were collapsed to preserve space (number of samples are indicated). DSV05 *=* DiatraeaSolisVargas05.

Because previous morphometric analyses did not improve the conventional approach for identification of *Diatraea* species, a fragment of CO II mitochondrial gene was amplified *in vitro* and studied to find a proper tool to assist in *Diatraea* identification. First, the phylogenetic relationships were inferred (Fig 2).

The CO II mitochondrial sequences discriminated the 4 species reared in laboratory conditions and the 3 species from field, grouping them in different clades which include some reported sequences in GenBank (*D*. *busckella*, *D*. *saccharalis*, *D*. *centrella*) [22]. It is important to mention that in our knowledge the sequences of CO II from *D*. *tabernella*, *D*. *indigenella* and *D*. *albicrinella* generated in this work constitute the first report, and they grouped in a consistent and separate manner from the other clusters in agreement with male genitalia morphology. In addition, according to the latest review on the taxonomic status of the *Diatraea* species in the Western Hemisphere [6], *D*. *centrella* and *D*. *albicrinella* constitutes new records for Colombia and DiatraeaSolisVargas05 is a new record for the world.

Currently, the species occur in nature as complexes conformed by different closely related groups which are very similar in morphological terms, but probably different in some physiological aspects [38]. Generally, the new populations that accomplish with such characteristics are recognized as cryptic species. In the case of *D*. *saccharalis*, the phylogenetic tree shows two different clades consistently separated. The first one includes 38 samples of reared insects (named for further analysis as *D*. *saccharalis* 1) and does not include any previously reported sequence. The other one contains 4 samples collected in field which grouped very close with a reported sequence from Brazil and sequences from Mexico, Venezuela, Jamaica and USA (named for further analysis as *D*. *saccharalis* 2). Interestingly, the geographical origins of both Colombian insect groups are different (Cauca Valley and Cundinamarca, respectively).

**Fig 3:**
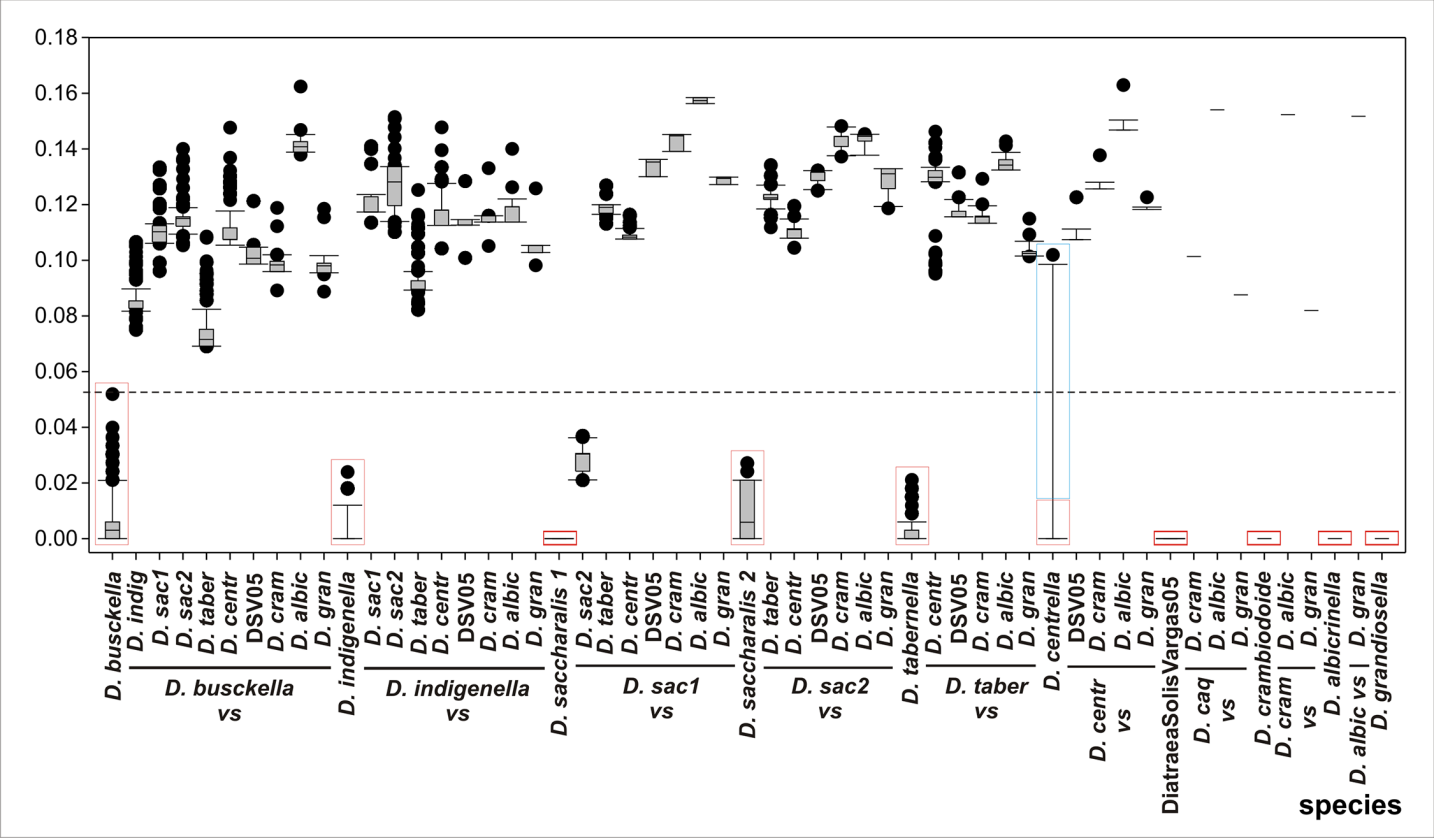
Pairwise phylogenetic distances. Intraspecific relationships are boxed with red rectangles and interspecies K2P distances were plotted as box plots. The boundary of the boxes closest to zero indicates the 25^th^ percentile, the line within the box marks the median, and the boundary of the box furthest from zero indicates the 75^th^ percentile. Error bars above and below the box indicate the 90^th^ and 10^th^ percentiles, respectively. Black filled circles indicate outlying points. Dashed line indicates a putative cut-off between intraspecific (including putative cryptic species) and interspecific variability without consideration of outlying points from *D*. *centrella* (AY320491.1) and *D*. *crambidoides* (AY320493.1) which are boxed within blue rectangles. *D*. *indig* = *D*. *indigenella; D*. *sac1* = *D*. *saccharalis* 1; *D*. *sac2* = *D*. *saccharalis* 2; *D*. *taber* = *D*. *tabernella*; *D*. *cram* = *D*. *crambidoides*; *D*. *gran* = *D*. *grandiosella; D*. *centr* = *D*. *centrella;* DSV05 *=* DiatraeaSolisVargas05.

To investigate CO II genetic distances that assist in the understanding of the genetic consistence relative to the current classification in species (including the putative cryptic species coded as *D*. *saccharalis* 1 and 2) a study of Kimura 2-parameter was carried out (Fig 3). Thus, this approach showed that the median intraspecific variability values ranged from 0.000 in *D*. *saccharalis* 1 or 0.002 in *D*. *indigenella* to 0.00354 in DiatraeaSolisVargas05, and they were always lower than the median values corresponding to interspecific variabilities, where the smallest distance occurred between *D*. *saccharalis* 1 and 2 (0.029) and the longest one occurred between *D*. *albicrinella* respect to *D*. *saccharalis 1* (0.169), *D*. *crambidoides* (0.168) and DiatraeaSolisVargas05 (0.165). Therefore, there was a correlation between morphological and genetic analysis based on genitalia and CO II, respectively.

Current *Diatraea* species were separated among themselves for distances greater than 0.07, highlighting that *D*. *tabernella* and *D*. *busckella* showed the closest relationship (0.078). In fact, the cladogram reflected a pattern coincident with this observation. Based on morphology of the male genitalia, the 5 specimens labelled in this paper as DiatraeaSolisVargas05, are part of an undescribed new species, closely related to *D*. *fuscella* described from Costa Rica (Solis, pers. comm.). This group showed a very low intraspecific variability and the CO II analysis showed a high consistency on belonging to the same clade. A similar situation occurred with one reported sequence of *D*. *centrella* from Ecuador (AY320491.1) which probably should be considered as a member of a cryptic species because it strongly increased the intraspecific variability of this species.

Specimens of *D*. *busckella* also showed the highest intraspecific diversity than the other species, and this result is consistent with the tree topography for this clade (Fig 2). Meanwhile, *D*. *saccharalis* represented another interesting case. Particularly, the variants here mentioned as *D*. *saccharalis* 1 and 2 had distances comprised in the range of intraspecific variability, with a possible scenario of the occurrence of cryptic species. Interestingly, both groups are very consistent and did not show overlapping in the ranges of distances established for each set. Differences among populations of *D*. *saccharalis* have been reported since 1990 when Pashley et al. [10] suggested that this species should be considered as two according to divergences verified in specimens collected in Brazil and USA. Other study based on CO II also reported two different groups for *D*. *saccharalis*, one including individuals from Mexico and South America and the other from southern USA [22]. Later, insects from Jamaica, USA (Texas and Florida), Mexico, Venezuela, Uruguay, Colombia and Brazil (Goiás, Mato Grosso, Paraná, Pernabuco, Sao Paulo) were analyzed showing that specimens collected in Colombia expressed the maximum divergence, which is consistent with our results [23]. A similar conclusion was obtained when pheromone extracts were studied [23]. Besides, studies of *D*. *saccharalis* diversity based on CO I and *Amplified fragment length polymorphism* (AFLP), and then with CO II, showed distances close to 0.02–0.03, similar to the values obtained in this work for *D*. *saccharalis* 1 and 2 [2, 9].

**Fig 4:**
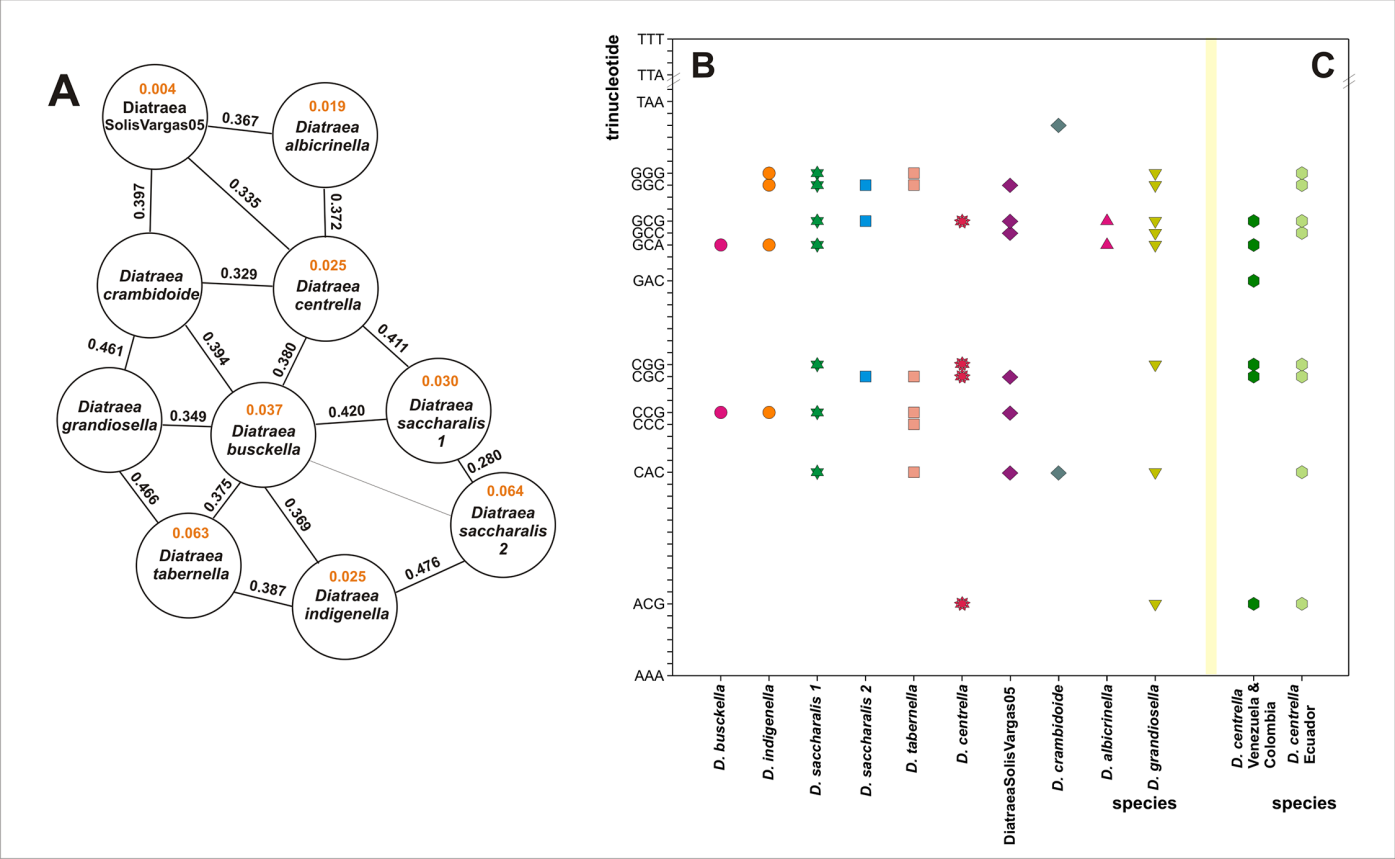
Relative abundance of trinucleotides for CO II. **A.** Distances between pairs of trinucleotide-based genetic signatures. Black numbers indicate interspecies distances and orange numbers indicate the average of intraspecific distances. *Diatraea saccharalis* 1 and 2 are analyzed as different phylogenetic groups (putative cryptic species). **B.** Differential patterns of trinucleotide absences in the sense strand of CO II fragment sequences from each analyzed clade. In the Y-axes are indicated only the two trinucleotide limits considered of the 64 possibilities (AAA and TTT) and all those which are absent in some sequences. **C.** Differential patterns of trinucleotide absences between the isolates of *D*. *centrella* from Venezuela (Vz) and Ecuador (Ec) [22]. DSV05 *=* DiatraeaSolisVargas05.

With the aim to add more genetic value to the *Diatraea* classification based on CO II sequence, the pattern of relationships between the observed and expected frequencies of trinucleotides was determined (Fig 4). As it is known, there are a total of 64 possibilities of combinations of nucleotides in sequences of length 3, but their occurrence into a particular genome segment constitutes a signature for that DNA sequence and a possible DNA bar code. This property is called “trinucleotide relative abundance” and can be used for species recognition [31].

This approach made it possible to find a particular pattern for each selected clade, both at the level of species and of other taxonomic groups such as cryptic species or variants. In this way, the discerning power at the taxonomic level offered by the CO II fragment is shown. In fact, the intraspecific variabilities among specimens (which average is 0.039 ± 0.018%) were lower than the interspecific relationships (which average is 0.396 ± 0.046% when *D*. *saccharalis* 1 and 2 are excluded), giving value to this different way of measuring distances between related groups. It is important to highlight that *D*. *saccharalis* 1 and 2 had distances between them (0.28) close to those obtained for the comparisons between pairs of species, thus giving solidity to the taxonomic recognition for these groups of specimens associated with particular geographic regions. Additionally, the CO II trinucleotide signature is clearly different (Fig 4B). A similar result was observed for the specimens of *D*. *centrella* from Colombia and Ecuador, showing that this observation at the sequence level can be transformed into an important element to complement the proper identification of field specimens.

### CO II analyses without sequencing

**Fig 5:**
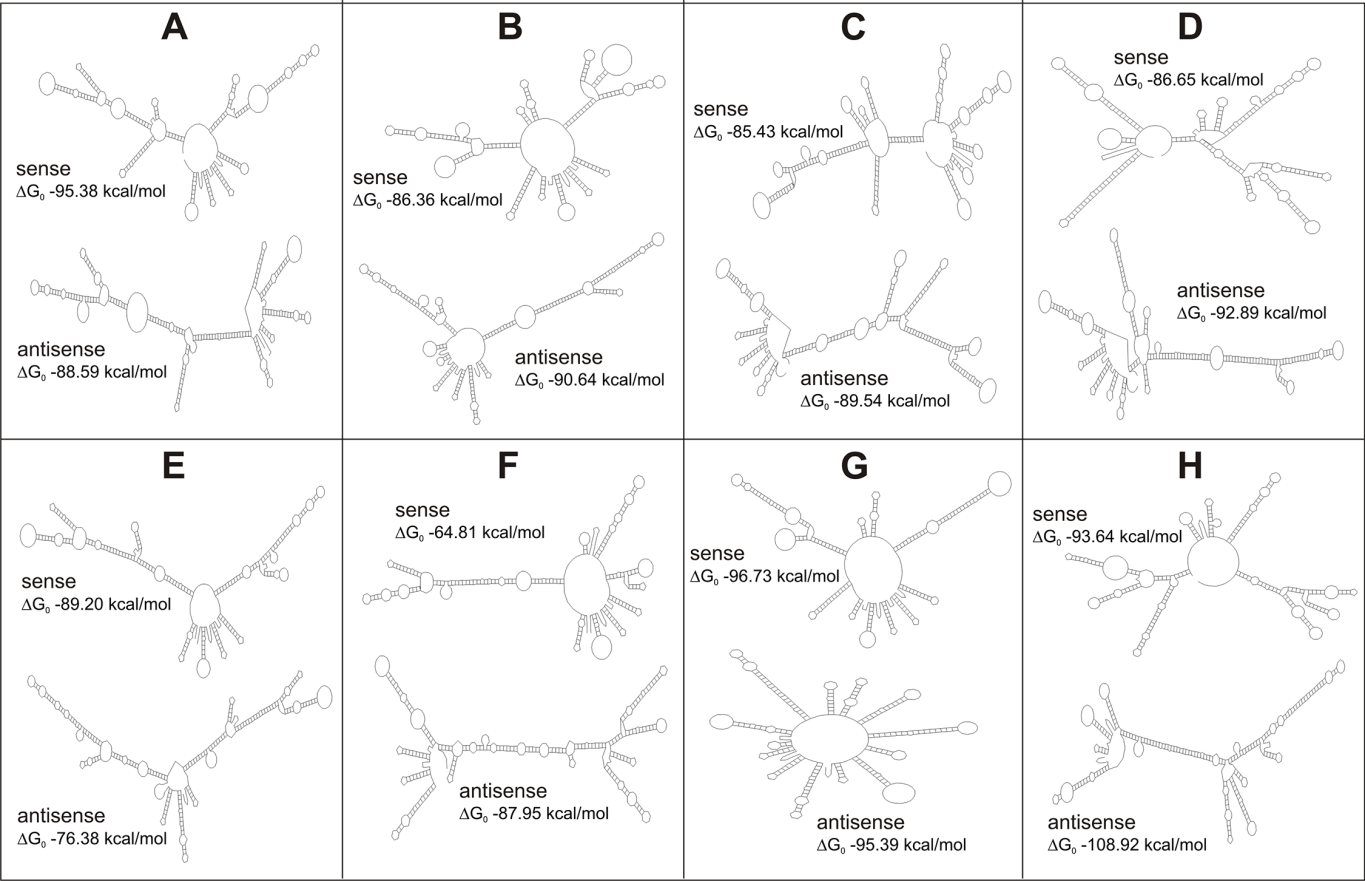
Single stranded DNA secondary structure prediction. The most favorable predicted sense and antisense conformers are shown. **A.** *D*. *busckella***. B.** *D*. *indigenella***. C.** *D*. *saccharalis* 1. **D.** *D*. *saccharalis* 2. **E.** *D*. *tabernella***. F.** *D*. *centrella***. G.** DiatraeaSolisVargas05. **H.** *D*. *albicrinella*.

Having confirmed that CO II gene sequences can be used to accurately classify *Diatraea* species (considering the genitalia-based key as a gold standard), a molecular method was developed that does not require sequencing of PCR fragments in order to accelerate the time to obtain results, as well as to increase the number of samples that can be analyzed. Thus, a PCR-SSCP methodology was selected for its high potential revealed in variability studies [39–41], and the results were compared with genitalia analyzes and bioinformatics studies based on CO II. In first place, the most favorable secondary structure of sense and antisense DNA strands from PCR products of CO II were predicted (Fig 5).

**Fig 6:**
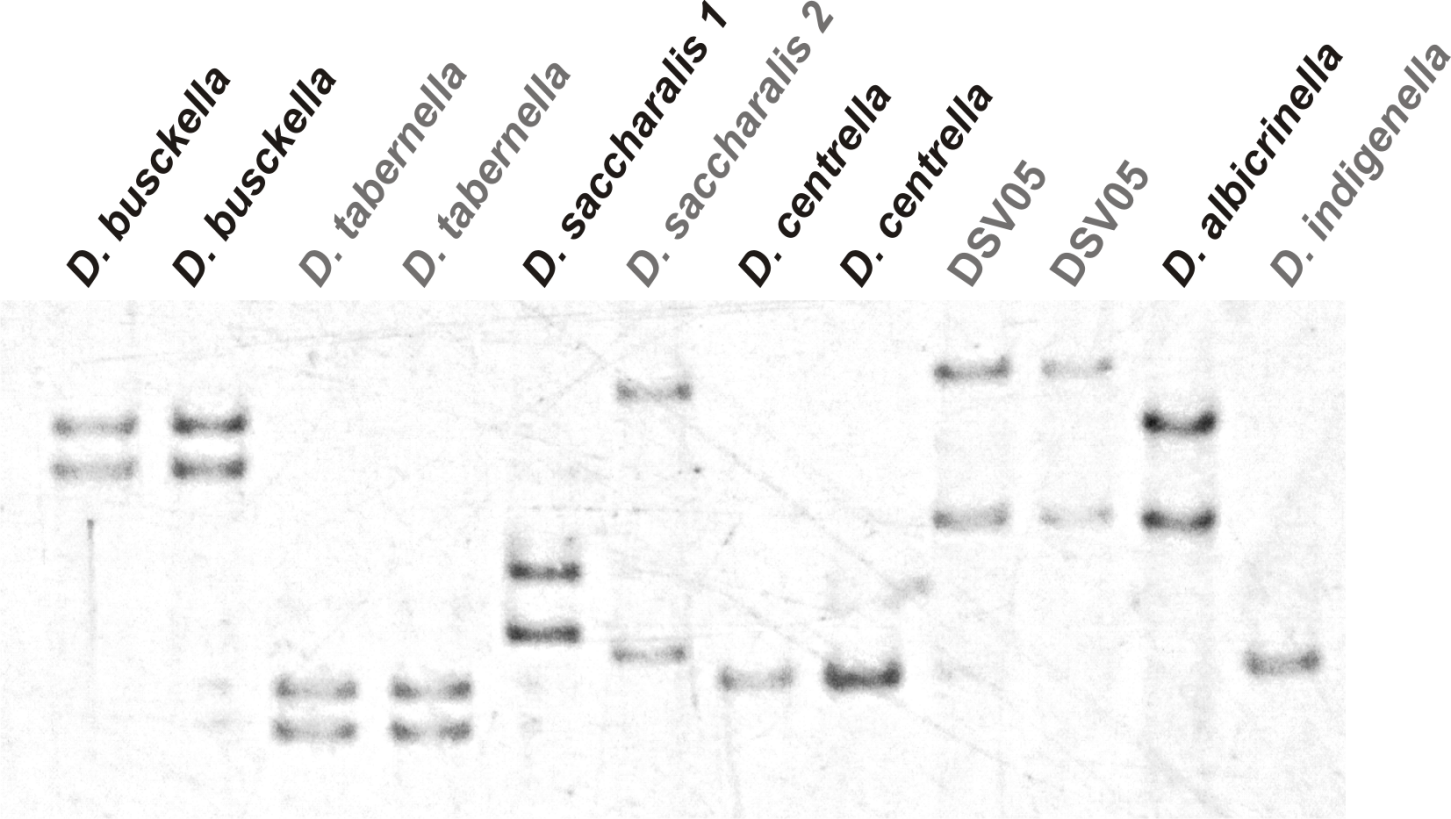
Differential patterns by SSCP of CO II PCR fragments. The photography corresponds to a 6% non-denaturing PAGE stained with the silver nitrate method, and shows the differential migration of some CO II amplified products obtained from different *Diatraea* spp. specimens previously classified by genitalia.

This analysis showed that all specimens could be identifiable since the conformers were sufficiently varied to migrate differentially in an electrophoresis. Taking in consideration that assumption, all individuals from the insect colonies and field samples were studied by the combination of CO II PCR and SSCP (Fig 6).

Notably, the different clades of *Diatraea* spp. were correctly differentiated by the SSCP analysis derived from the CO II amplification products according to the conformer predictions (Fig 5) and the distance analyses (Figs 3 and 4). This methodological approach illustrated its potential to initiate the characterization of cryptic species by distinguishing individuals classified as *D*. *saccharalis* 1 and 2. In this way, the classification of samples for this invertebrate, regardless of genus, state or instar, can be simplified by PCR of CO II mitochondrial gene and its subsequent electrophoretic analysis.

**Fig 7:**
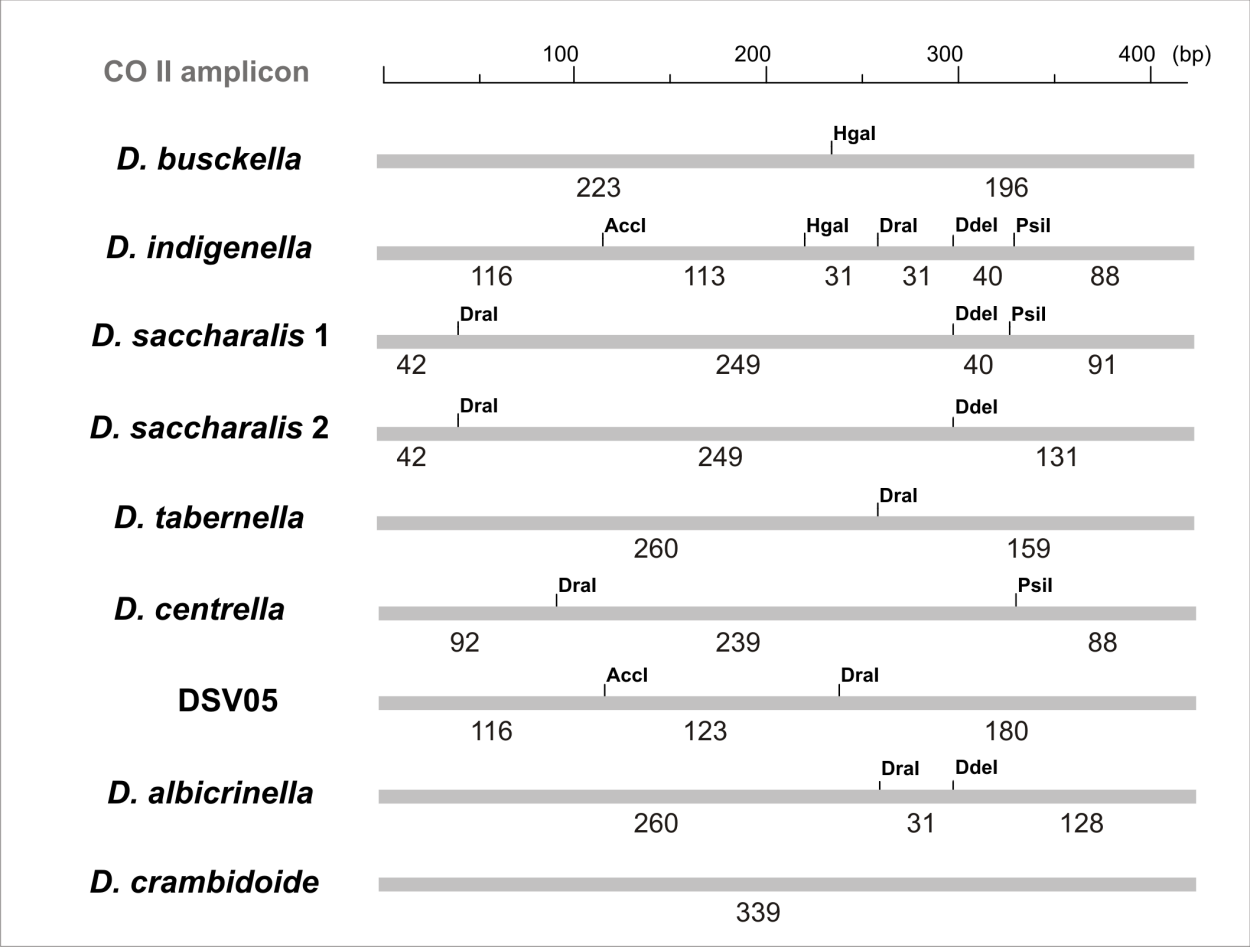
Species-specific restriction patterns for CO II PCR product. The distribution of targets sites for a set of 5 DNA endonucleases (AccI, HgaI, DraI, DdeI and PsiI) is shown. Below the thick grey line the expected size of each fragment is indicated, measured in base pairs (bp).

We also propose that it may be possible to treat the PCR products (419–422 bp) with restriction endonucleases, but we did not evaluate this experimentally. PCR-RFLP (Polymerase Chain Reaction—Restriction Fragment Length Polymorphism) remains an eligible methodology for the analysis of genetic diversity without sequencing [42, 43]. For this purpose, the *in silico* differential pattern of each taxonomic group is shown (Fig 7).

Thus, a treatment with AccI and HgaI would be useful to differentiate *D*. *busckella* (196 bp, 223 bp), *D*. *indigenella* (113 bp, 116 bp and 190 bp) and DiatraeaSolisVargas05 (116 bp and 303 bp). If the amplicon is not digested (the case for *D*. *tabernella*, *D*. *centrella*, *D*. *albicrinella* and *D*. *sacharallis* specimens) a subsequent treatment with *Dra*I and *Dde*I would collaborate in the proper identification [*D*. *tabernella* (159 bp and 260 bp), *D*. *centrella* (92 bp and 327 bp), *D*. *albicrinella* (31 bp, 128 bp and 260 bp) and *D*. *sacharallis* (42 bp, 131 bp and 249 bp)]. Finally, a further treatment with *Dra*I and *Psi*I for the *D*. *saccharalis* amplicons would allow to identify them as belonging to the Group 1 (42 bp, 91 bp and 289 bp) or 2 (42 bp and 380 bp). Because more experimental steps and availability of a greater amount of PCR products are required, the SSCP methodology becomes more robust and sensitive since it does not involve treatments with other enzymes and requires little amount of DNA.

## Conclusions

Male genitalia analysis is a laborious method, which requires the dissection of adult individuals and their observation in a stereo microscope. In addition, the number of animals available for observations may be limited by natural enemies occurring in the field. In this work, male genitalia observation has allowed the identification of seven species of *Diatraea* in Colombia: *D*. *busckella*, *D*. *tabernella*, *D*. *indigenella*, *D*. *saccharalis*, *D*. *centrella*, DiatraeaSolisVargas05 and *D*. *albicrinella*; from which the latter three constitutes new record for the species in the country.

The morphometric analysis carried out suggests that comparisons of male genitalia’s size and shape are not an accurate method for species identification in populations of *Diatraea* spp. This was also suggested previously by Mutanen et al 2005 [44], concluding that genital morphology is not constant and should therefore be used with caution in lepidopteran taxonomy. In spite of this, we are proposing ranges of dimensions on the genitalia that can assist in conflicting cases of identification.

An approach based on CO II mitochondrial gene was performed. CO II sequence analyses offer the possibility to classify specimens belonging to *Diatraea* with a similar power of discrimination than genitalia approach, but with the advantage of working with individuals of any sex and state (i.e., larva, pupa or adult). SSCP method is an adequate screening technique, recommended as a first approach, useful to classify in a relatively rapid way numerous individuals. If a sample were to reveal a new SSCP pattern, not associable with any recognized taxonomical group (for this It is important to have positive controls for each species or recognized taxonomical groups), the suggested way would be continuing with a PCR assay using the primers having M13 sequences and a subsequent Sanger sequencing. Then, a phylogeny inference and the identification of a trinucleotide signature can collaborate in the allocation in a recognized clade, or in the proposal of a new one. We consider that this workflow would be useful to assist in a continental-wide study to aiming recognize the *Diatraea* species. Besides, until crossbreeding results are obtained, it is important to determine a limit for genetic distance values for the recognition of cryptic species. As previously described by Joyce et al., [9], distances close to 0.020–0.030 for genes such as CO I or CO II could be considered appropriate ranges for this kind of assumption. In consequence, the populations mentioned here as *D*. *saccharalis* 1 and 2 could be considered as cryptic species occurring in Colombia.

An update on the taxonomical status of the sugarcane stem borers in the Americas will allow the establishment of proper biological control programs reducing the economic impact of these pests on projects related to panela (unrefined whole sugarcane sugar), a product of recognized impact on food security in Central and South America, and other projects expanding on the production of sugar, energy and ethanol.
